# Apparent tunneling barrier height and local work function of atomic arrays

**DOI:** 10.3762/bjnano.9.283

**Published:** 2018-12-17

**Authors:** Neda Noei, Alexander Weismann, Richard Berndt

**Affiliations:** 1 Institut für Experimentelle und Angewandte Physik, Christian-Albrechts-Universität zu Kiel, 24098 Kiel, Germany

**Keywords:** scanning tunneling microscopy, tunneling barrier height, work function

## Abstract

Spatially resolved measurements of the apparent tunneling barrier height Φ_app_ in scanning tunneling microscopy have been used to estimate variations of the local work function Φ of surface structures. We experimentally show that Φ_app_ can fail as a measure of Φ. The discrepancies are attributed to a kinetic-energy contribution to Φ_app_. This contribution depends on the lateral extent of the tunneling current filament and, consequently, on the local surface structure.

## Findings

The work function of a metal surface [1], the work required at temperature *T* = 0 K to move an electron from the metal to infinity, is relevant for, e.g.,the behavior of electronic devices [2], the energetics of molecules at surfaces [3], including reactions [4–5], and electronic states confined near surfaces [6]. Practical surfaces are finite in size and contain plenty of inhomogeneities that cause local variations of the electrostatic potential. This is reflected by the notion of a local work function Φ [7–9]. Adsorbates modify Φ in an intriguing manner [10–13]. In turn, variations of Φ produce, e.g., atomic-scale contrast in field-emission microscopy, photo-emission electron microscopy, and low-energy electron microscopy [14–16].

Atomic steps are a well-known example of a structure that affects the local work function Φ. The spill-out of electrons from a planar crystal surface creates a dipole layer that increases Φ [17–18]. Steps modify the spill-out and thus reduce Φ [19] as observed by Kelvin probe measurements [20] and photoelectron spectroscopy [9].

The concept of a local work function is particularly relevant for scanning tunneling microscopy [7–8]. In the most simple one-dimensional model the exponential variation of the tunneling current *I* at low bias with the tip–sample distance *z* is directly related to the average of the work function 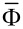 of the tip and the sample [21–22]. A closer look shows that factors like the image potential of the tunneling electron or the electronic structure of the electrodes complicate matters [23–26]. *I* still varies exponentially with *z*, but 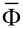 is replaced with an apparent barrier height Φ_app_ [27]. Although there is no simple expression connecting Φ_app_ and the local work function of the sample Φ (partially because the tip structure is usually unknown), it is common practice to assume that measured variations of Φ_app_ represent those of Φ [28–31].

Here we experimentally show that Φ_app_ can fail as a measure of Φ. *I*(*z*) data from Cu atoms arranged in extended single-atom rows and double rows as well as from trenches of single-atom depth are analyzed. Φ_app_ determined from these data varies significantly, but the variation is opposite to the expected trend of the local sample work function. We explain this observation in terms of the lateral extent of the tunneling-current filament. While an atomic asperity at the sample is expected to exhibit a reduced Φ, it simultaneously reduces the lateral range over which a significant part of the current flows. This effectively raises the energy required to overcome the potential barrier between the tip and the sample.

In the context of the scanning tunneling microscope, the idea of an increased barrier due to lateral confinement has been attributed to J. Tersoff in [27]. Lateral confinement plays a key role in ballistic transport through nanoscale constrictions [32] and was suggested to affect the apparent barrier height in single-atom contacts [33]. Atomistic transport calculations have been performed for Au contacts in [34]. Symmetric junctions comprised of two (001) surfaces, either planar or with an adatom or with a five-atom pyramid, were considered. The apparent barrier heights extracted were 5.7, 6.7, and 6.8 eV, respectively. Because of a systematic error of the local density approximation used in the calculations [34], the value of the flat surface is larger than the experimental one [35]. However, the trend of a barrier height increase with decreased lateral size of the tunneling-current path is expected to be reliable. These results predict a kinetic energy contribution of the order of 1 eV for tunneling between two atomically sharp structures.

Experiments were performed in an ultrahigh vacuum STM operated at 4.5 K. Cu(111) surfaces were cleaned by Ar^+^ sputter/anneal cycles. W tips were electrochemically etched from polycrystalline wire. After sputtering, they were further prepared in situ by indenting them into the Cu(111) substrate. Because of this procedure, the tips were presumably covered with Cu.

**Figure 1:**
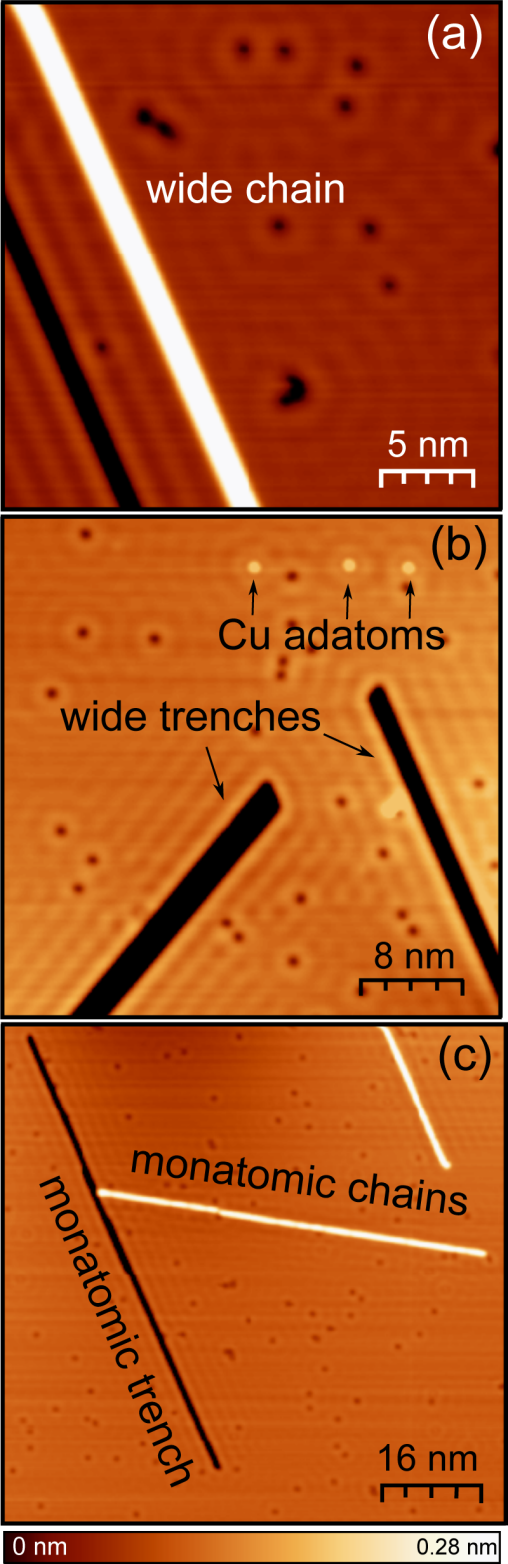
STM images of a Cu(111) surface with chains and trenches of monolayer height. (a) A Cu chain approximately two atoms wide and a parallel trench. (b) Two wide trenches (approx. 2 and 3 atoms) with deposited single Cu atoms. (c) Monatomic chains and trenches. The images were acquired at (a) *V* = 20 mV and *I* = 3 nA and (b, c) *V* = 100 mV and *I* = 5 nA.

In surface areas close to the point of indentation (typical distance 200 nm) long, straight atomic chains are formed on the substrate (Figure 1). While the width of the chains varies, one- and two-atom wide chains are frequently observed. They may be identified from their apparent widths in topographs and from a characteristic signature in differential conductance (d*I*/d*V*) spectra. As first reported by Fölsch et al., short single-atom Cu chains on Cu(111) exhibit an unoccupied resonance at 1.5 eV above the Fermi energy *E*_F_ [36]. Closely related data including the limit of very long chains were reported from chains on Ag(111) [37]. In addition to chains, we occasionally observed long trenches of varying widths with an apparent depth of one atomic layer or less (Figure 1). Finally, single Cu adatoms were prepared by transfer from the tip to the surface [33].

**Figure 2:**
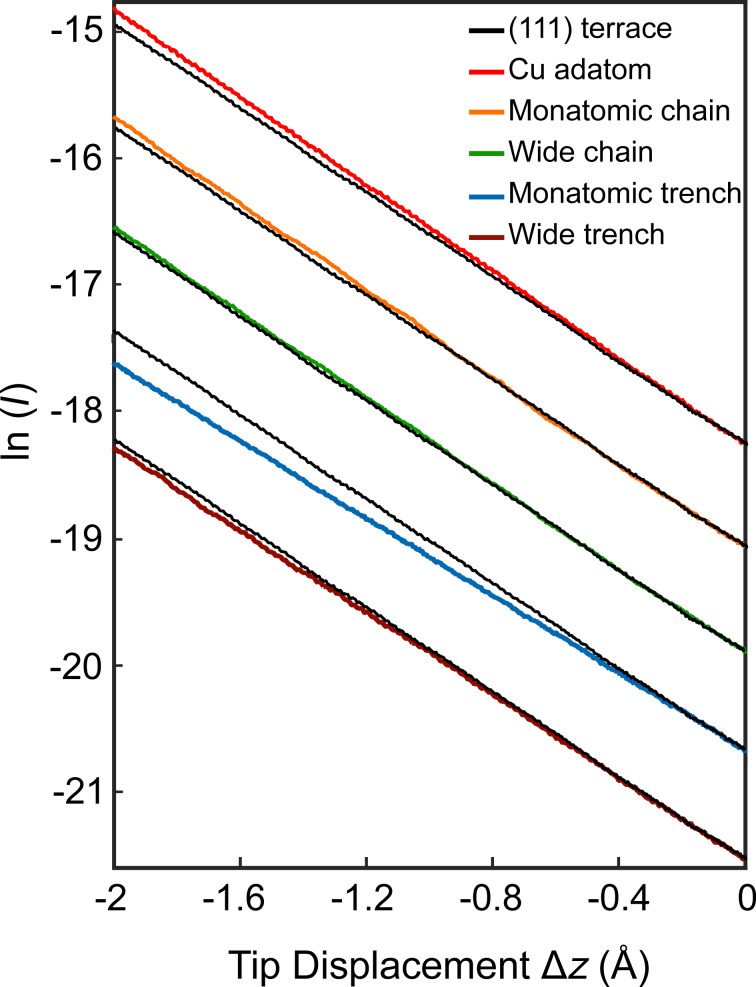
Current–distance data *I*(Δ*z*). Negative values of Δ*z* indicate reduced tip–sample distances with respect to the initial separation defined by *I* = 200 pA and *V* = 20 mV. Data recorded from a (111) terrace (black), a Cu adatom, a monatomic chain, a wider chain (approx. 2 atoms), and trenches of monolayer depth with single-atom and wider width (approx. 2 atoms) are displayed. The curves are arbitrarily offset along the ordinate for clarity. The terrace data are shown with each curve for comparison. Small undulations of the data are due to low-frequency vibration of the microscope.

To measure the current *I* as a function of the vertical tip excursion Δ*z* the feedback loop of the STM was disabled at a sample voltage of *V* = 20 mV and a current of *I* = 200 pA. The tip was then brought closer to the structure under investigation at a rate of 1.7 nm/s while recording *I*. Figure 2 shows typical results from a clean (111) terrace, a Cu adatom, a monatomic chain, a wide chain, and grooves of monolayer depth with single-atom or wider width. There are systematic differences between the slopes of the terrace data (black line) and the other data sets (colors). On asperities such as adatoms and narrow chains the current varies more rapidly, on narrow trenches the slope is reduced. The slopes observed on wide trenches and chains are close to the terrace data.

The apparent barrier heights Φ_eff_, which we extracted from the exponential variation of the current 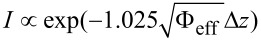, are presented in Table 1. These values were measured with the same tip apex. The absolute values are obtained using the calibration of the piezoelectric transducer, which leads to an estimated uncertainty of 10%. The ratios between them, however, are not affected. The maximal apparent barrier height is measured on Cu adatoms. It exceeds the minimal value, which is observed from monatomic trenches, by approx. 0.9 eV and is 0.3 eV larger than on flat terraces. Repetition of the measurements at another low bias voltage (*V* = 10 mV) led to virtually identical results as expected.

**Table 1:** Apparent barrier heights Φ_app_ extracted from Figure 2.

structure	Φ_app_ (eV)

(111) terrace	3.9
adatom	4.2
monatomic chain	4.2
wide chain	4.0
monatomic trench	3.3
wide trench	3.8

The electron spill-out at steps is associated with a dipole moment [19]. It counteracts the dipole present at flat surfaces and therefore reduces the work function. Atomic chains and single atoms represent stronger corrugations of a surface and therefore are expected to further enhance this effect. The observed trend of Φ_app_, however, is opposite to this expectation.

Φ_app_ may vary when the local surface normal is at an angle α with the *z* direction of the STM [38]. However, this is expected to reduce Φ_app_ by a factor cos^2^α and thus cannot explain our large experimental values. In any event, we measured Φ_app_ of atoms and chains on maxima of the topographies, where α ≈ 0.

We therefore attribute the peculiar variation of Φ_app_ at adatoms and chains to the kinetic-energy contribution that was proposed in earlier theoretical works [27,33–34]. Considering the topographies of adatoms and chains, the lateral extension of the tunneling current filament is expected to be narrower on these structures than on flat terraces. The trend of the experimental Φ_app_ data is consistent with these expectations and the theoretical results of [34]. A quantitative estimate of the kinetic-energy contributions has to take into account the local work function of the structures under investigation. Unfortunately, neither experimental nor theoretical data are currently available for the structures of Figure 1.

The data from trenches require an additional consideration. When the tip is centered above a trench, a part of the tunneling current is expected to flow to the edge atoms of the adjacent terraces. Indeed, the apparent depth of single-atom trenches is significantly smaller than a step height. On one hand, this contribution to the current widens the current filament. On the other hand, it is not perpendicular to the surface normal and consequently the geometric factor introduced above may play a role and reduce Φ_app_.

In conclusion, topographic features determine the extent of the electron wave function across the tunneling path in the STM and thus add a kinetic energy contribution to the apparent barrier height Φ_app_. The difference of this contribution between atomic protrusions and trenches is of the order of 1 eV. This complicates the relation between the local sample work function Φ and the measured Φ_app_. The shape of the tip also influences the degree of confinement and presumably contributes to the scatter of Φ_app_ observed with different tips.

## References

[1] Derry G N, Kern M E, Worth E H (2015). J Vac Sci Technol, A.

[2] Kahn A (2016). Mater Horiz.

[3] Jiang Y, Li J, Su G, Ferri N, Liu W, Tkatchenko A (2017). J Phys: Condens Matter.

[4] Lang N D, Holloway S, Nørskov J K (1985). Surf Sci.

[5] Vayenas C G, Bebelis S, Ladas S (1990). Nature.

[6] Fischer R, Schuppler S, Fischer N, Fauster T, Steinmann W (1993). Phys Rev Lett.

[7] Binnig G, Rohrer H, Gerber C, Weibel E (1982). Phys Rev Lett.

[8] Binnig G, Rohrer H (1983). Surf Sci.

[9] Wandelt K (1997). Appl Surf Sci.

[10] Michaelides A, Hu P, Lee M-H, Alavi A, King D A (2003). Phys Rev Lett.

[11] Ploigt H-C, Brun C, Pivetta M, Patthey F, Schneider W-D (2007). Phys Rev B.

[12] Bagus P S, Käfer D, Witte G, Wöll C (2008). Phys Rev Lett.

[13] Roman T, Groß A (2013). Phys Rev Lett.

[14] Müller E W (1937). Z Phys.

[15] Bauer E (1998). Surf Rev Lett.

[16] Rotermund H H (1997). Surf Sci Rep.

[17] Seitz F (1940). Modern Theory of Solids.

[18] Lang N D, Kohn W (1971). Phys Rev B.

[19] Smoluchowski R (1941). Phys Rev.

[20] Besocke K, Krahl-Urban B, Wagner H (1977). Surf Sci.

[21] Simmons J G (1963). J Appl Phys.

[22] Simmons J G (1963). J Appl Phys.

[23] Binnig G, Garcia N, Rohrer H, Soler J M, Flores F (1984). Phys Rev B.

[24] Becker M, Berndt R (2010). Phys Rev B.

[25] Becker M, Berndt R (2010). Appl Phys Lett.

[26] Pitarke J M, Echenique P M, Flores F (1989). Surf Sci.

[27] Lang N D (1988). Phys Rev B.

[28] Jia J F, Inoue K, Hasegawa Y, Yang W S, Sakurai T (1998). Phys Rev B.

[29] Sasaki M, Yamamoto S (2007). Shinku (1958-2007).

[30] Herz M, Schiller C, Giessibl F J, Mannhart J (2005). Appl Phys Lett.

[31] Altenburg S J, Berndt R (2014). New J Phys.

[32] Ihn T (2009). Semiconductor Nanostructures.

[33] Limot L, Kröger J, Berndt R, Garcia-Lekue A, Hofer W A (2005). Phys Rev Lett.

[34] Garcia-Lekue A, Wang L W (2010). Phys Rev B.

[35] Fauster T, Steinmann W, Helvi P (1995). Two-photon photoemission spectroscopy of image states. Photonic Probes of Surfaces.

[36] Fölsch S, Hyldgaard P, Koch R, Ploog K H (2004). Phys Rev Lett.

[37] Sperl A, Kröger J, Néel N, Jensen H, Berndt R, Franke A, Pehlke E (2008). Phys Rev B.

[38] Binnig G, Rohrer H (1986). IBM J Res Dev.

